# First report demonstrating the presence of *Toxocara* spp. eggs on vegetables grown in community gardens in Europe

**DOI:** 10.1016/j.fawpar.2022.e00158

**Published:** 2022-04-20

**Authors:** Sara R. Healy, Eric R. Morgan, Joaquin M. Prada, Martha Betson

**Affiliations:** a University of Surrey, School of Veterinary Medicine, Daphne Jackson Road, Guildford, Surrey GU2 7AL, UK; b Queen's University, Institute for Global Food Security, Biological Sciences Building, 19 Chlorine Gardens, BT9 5DL Belfast, UK

**Keywords:** *Toxocara*, Food safety, Vegetables, Community gardens, Allotments, Zoonosis, Public health

## Abstract

*Toxocara canis* and *T. cati* are zoonotic roundworm parasites of dogs, cats and foxes. These definitive hosts pass eggs in their faeces, which contaminate the environment and can subsequently be ingested via soil or contaminated vegetables. In humans, infection with *Toxocara* can have serious health implications. This proof-of-concept study aimed to investigate the presence of *Toxocara* spp. eggs on ‘ready-to-eat’ vegetables (lettuce, spinach, spring onion and celery) sampled from community gardens in southern England. The contamination of vegetables with *Toxocara* eggs has never been investigated in the UK before, and more widely, this is the first time vegetables grown in community gardens in Europe have been assessed for *Toxocara* egg contamination. Sixteen community gardens participated in the study, providing 82 vegetable samples fit for analysis. Study participants also completed an anonymous questionnaire on observed visits to the sites by definitive hosts of *Toxocara*. Comparison of egg recovery methods was performed using lettuce samples spiked with a series of *Toxocara* spp. egg concentrations, with sedimentation and centrifugal concentration retrieving the highest number of eggs. A sample (100 g) of each vegetable type obtained from participating community gardens was tested for the presence of *Toxocara* eggs using the optimised method. Two lettuce samples tested positive for *Toxocara* spp. eggs, giving a prevalence of 2.4% (95% CI =1.3–3.5%) for vegetable samples overall, and 6.5% (95% CI = 4.7–8.3%; *n* = 31) specifically for lettuce. Questionnaire data revealed that foxes, cats and dogs frequently visited the community gardens in the study, with 88% (68/77) of respondents reporting seeing a definitive host species or the faeces of a definitive host at their site. This proof-of-concept study showed for the first time the presence of *Toxocara* spp. eggs on vegetables grown in the UK, as well as within the soil where these vegetables originated, and highlights biosecurity and zoonotic risks in community gardens. This study establishes a method for assessment of *Toxocara* spp. eggs on vegetable produce and paves the way for larger-scale investigations of *Toxocara* spp. egg contamination on field-grown vegetables.

## Highlights

• First report of *Toxocara* eggs on vegetables grown in community gardens in Europe.
• *Toxocara* also detected in the soil where these vegetables originated.
• Definitive hosts for *Toxocara canis* and *T. cati* frequently visit community gardens.
• Sedimentation method recovered the most *Toxocara* eggs from spiked lettuce samples.

## 1 Introduction

*Toxocara* canis and *T. cati* are zoonotic roundworm parasites which reside in the intestines of infected dogs, cats and foxes. Adult worms produce a large number of eggs which are subsequently shed in the faeces of these definitive hosts (Morgan et al., 2013). Once in the environment, eggs develop to become infective and can persist in the soil for long periods (Fan et al., 2013). *Toxocara* can only complete its lifecycle within a definitive host, but alternative species, including humans, can become infected by ingestion of eggs or larvae, leading to toxocariasis. Within these ‘accidental’ hosts, the parasite cannot complete its maturation, but larvae migrate around the body to various organs. In some cases, this can result in severe pathology with debilitating clinical consequences for humans including ophthalmic and neurological disorders (Ma et al., 2018).

Whilst it is widely accepted that contaminated soil acts as a source of *Toxocara* eggs with potential to infect animals and humans, less is known about food-borne transmission of this parasite. *Toxocara* spp. eggs have been found to contaminate several different vegetable types (Klapec and Borecka, 2012; Healy et al., 2021), which could pose a risk to humans if consumed unwashed. However, this transmission route has received relatively little attention in the literature, especially in relation to small-scale production of vegetables on public land. Community gardens, also known as allotments, are small plots of land, often publicly owned, which are rented by individuals at nominal rates for growing crops or flowers. They provide a way for consumers to grow their own vegetables, bringing many reported benefits to human health and wellbeing (Dobson et al., 2021), and are increasing in popularity in many EU countries (van der Jagt et al., 2017). Gardens are typically accessible to wildlife species such as foxes and free-roaming cats (pet or stray), while pet dogs might also be permitted access. Carnivore faeces have been frequently observed in kitchen gardens and shown to contain pathogens including *Toxocara* spp. as well as *Echinococcus multilocularis* and *Toxoplasma gondii* (Poulle et al., 2017), with evidence of transfer to soil (Umhang et al., 2017). As such, these locations provide a highly relevant environment to assess the presence of *Toxocara* spp. eggs, with the close proximity of definitive host species, soil and growing crops enhancing the potential for contamination of food products. The aim of this proof-of-concept study was to assess a range of vegetable types grown in community gardens for the presence of *Toxocara* sp. eggs.

## 2 Materials and methods

### 2.1 Study population and vegetable sampling

Sixteen community gardens in the UK counties of Surrey and London were recruited into the 12-week study via direct electronic communication with the site managers. Site locations were selected based on their proximity to the testing laboratory to facilitate weekly sample collection and transportation. Six of the sites were located in semi-rural areas and ten sites were situated within residential localities. Plot holders at the community gardens were provided with a study information sheet and researcher contact information before project commencement. Large plastic crates were positioned at each site and checked for donations weekly, with plot holders asked to provide samples of lettuce, spinach, spring onions or celery grown on-site. These vegetable types were selected as ‘ready to eat’ and often consumed without peeling or cooking, so could pose a higher risk for consumers if not washed before ingestion. Participants were also asked to complete an anonymous questionnaire survey and include it with their vegetable donation, stating any sightings of definitive hosts or faeces on-site, and use of coverings/nets over crops. A total of 86 vegetable samples were donated between May 2021–July 2021 from 77 plot holders. Four samples were mouldy on receipt, hence not processed. Some donations received were of ready-to-eat vegetable types not originally requested, these included Swiss chard (*Beta vulgaris*), pak choi (*Brassica rapa* subsp. *Chinensis*) and garden mint (*Mentha spicata*). These were processed in the same manner as the other vegetable samples.

### 2.2 Toxocara spp. egg recovery

There are a number of published methods to analyse vegetable material for presence of parasitic eggs. In order to determine the most effective method to detect *Toxocara* spp. eggs on vegetables for this study, a series of experiments was undertaken using greenhouse-grown lettuce samples of 100 g, spiked with known quantities of *Toxocara* eggs (20, 50, 100 or 200), plus non-spiked negative controls. To obtain the eggs, adult *T. canis* worms were removed from the small intestines of foxes at post-mortem and eggs harvested from females as outlined by Pineda et al. (2021), followed by three washings in reagent grade water and resuspension in 1× phosphate buffered saline (PBS), pH 7.3. The suspended eggs were enumerated by manual count in two 10 μl aliquots by light microscopy, and the average calculated. Suspensions of known egg densities were prepared, and 25 μl volumes were added directly to the lettuce leaves in 3 locations and left to dry at room temperature for 2 h before being processed.

Three assays were tested by processing lettuce leaf samples as follows:

1) Two cycles of homogenisation in 200 ml glycine buffer (pH 5.5, 1 M), each of 30 s duration at 300 rpm within sterile paddle-beater bags with integrated 200 μm pore diameter filter strips, a method modified by Lalle et al. (2018). Liquid passing through the filter strip was collected and centrifuged at 2000 x*g* for 15 min at 4 °C to obtain a pellet for analysis.
2) A sequential sieving approach modified from Malkamäki et al. (2019), in which spiked samples were washed in beakers containing 500 ml water. This water was subsequently filtered through two layers (folded gauze in a funnel followed by a 38 μm metal sieve). The metal sieve was rinsed using 200 ml water, and the rinsate centrifuged at 2000 ×*g* for 5 min to obtain a pellet for analysis.
3) A sedimentation assay modified from Hajipour et al. (2021), in which spiked samples were washed in beakers containing 500 ml of physiological saline (0.95% NaCl), followed by overnight sedimentation and subsequent concentration of the sediment by centrifugation at 2000 ×*g* for 5 min. The pellets underwent NaCl flotation for one hour at room temperature, and the top 20 ml of flotation solution aspirated for further analysis.

A 5 ml aliquot was analysed using a McMaster counting chamber (Vetlab Supplies, UK), and the remaining fluid was further concentrated to obtain a pellet for subsequent molecular analysis using a method by Mes et al. (2001). Purified water was added to the top of each tube, followed by centrifugation at 4000 *g* for 5 min.

For each assay, the pellets obtained were assessed for the presence of *Toxocara* eggs by analysing 2 × 10 μl aliquots visually by light microscopy at 100× and 400× total magnification.

### 2.3 Molecular detection

In order to explore the threshold for molecular detection of *Toxocara* spp. eggs on vegetables, and to establish whether differentiation between *T. canis* and *T. cati* eggs could be achieved, the above egg-spiking experiment was repeated using the sedimentation method. A range of egg concentrations (5, 10, 20, 50 or 100) were spiked onto 100 g lettuce samples. The pellets obtained were heated at 90 °C for 10 min to promote egg disruption, as described by Tyungu et al. (2020) before DNA extraction using Powersoil kits (Qiagen, USA) according to manufacturer instructions with 10 min of full-power bead beating in a TissueLyser LT (Qiagen, USA).

Extracted DNA was subjected to qPCR testing as described by Tyungu et al. (2020), using a C1000 Touch 96-well RT-PCR machine (Biorad, USA) with previously published probes for *T. canis* (5’-FAM-CCATTACCACACCAGCATAGCTCACCGA-3’-NFQ-MGB) and *T. cati* (5-HEX-TCTTTCGCAACGTGCATTCGGTGA-3’-NFQ-MGB)] and forward primers [*T. canis* (5’-GCGCCAATTTATGGAATGTGAT-3′) and *T. cati* (5’-ACGCGTACGTATGGAATGTGCT-3′)] and shared reverse primer 5’-GAGCAAACGACAGCSATTTCTT-3′) for both *Toxocara* species (Tyungu et al., 2020). All reactions were performed in a total volume of 20 μl containing 1× SsoAdvanced Universal Probes Supermix (BioRad, USA), 2 μl template DNA, 0.5 μM of each primer and 0.25 μM of each labelled probe. Samples were tested in duplicate and run at 95 °C for 3 min followed by 95 °C for 10 s and 60 °C for 30 s for a total of 39 cycles. *T. canis* and *T. cati* DNA extracted from adult worms were used as positive controls, and no template used for negative controls. A sample was considered positive if there was detectable DNA at or before a cycle threshold of 38, as per Tyungu et al. (2020).

### 2.4 Sample processing

For the vegetable samples obtained from community gardens, weighed samples (100 g) of each vegetable type were analysed for the presence of *Toxocara* spp. eggs using the sedimentation method outlined above (method 3). Eggs were identified morphologically as previously described (Thienpont et al., 1979), with dimensions verified by graticule measurement. Due to similarities between the morphology of *T. canis* and *T. cati* eggs, differentiation is not possible by direct visualisation. Pellets obtained were subjected to DNA extraction and qPCR as outlined previously.

At least 100 g of the surrounding soil had also been provided for eight vegetable samples, which was tested for the presence of *Toxocara* spp. eggs using a method modified from Tyungu et al. (2020). In brief, 100 g of soil was sieved to remove large debris before being washed three times in 0.1% Tween 20. Samples were then mixed with enough saturated sodium nitrate solution (spg 1.30) to form a meniscus at the opening of the tube with a coverslip in place. Samples were left at room temperature overnight, before analysis of the coverslips by light microscopy at 10× and 40× objective. Eggs were again identified morphologically, with size verification by graticule measurement.

### 2.5 Statistical analysis

Prevalence and its 95% confidence intervals were calculated using the EpiTools software (Sergeant, 2018). For statistical analysis, GraphPad QuickCalcs Web site (https://graphpad.com/quickcalcs/contingency2/) was employed. *P*-values were calculated using Fisher's exact test. A P-value <0.05 was considered statistically significant.

## 3 Results

The sedimentation method was found to recover the highest number of *Toxocara* spp. eggs from spiked lettuce samples in this study, with three eggs detected by microscopic analysis for the 200-egg spike, and one egg visualised for the 50-egg spike. The homogenisation method only resulted in the detection of one egg on lettuce spiked with 200 eggs. However, no eggs were detected using the sequential sieving method for any of the spiked lettuce samples.

The qPCR analysis of the pellets obtained from spiked lettuce samples using the sedimentation method showed positive results for the 20, 50 and 100 egg spikes, as shown in Table 1. The primers selected were able to differentiate between the DNA of *T. canis* and *T. cati* in the positive control samples, and the eggs used for spiking experiments were found to be *T. canis*. Positive and negative control samples behaved as expected.

**Table 1:** Results of qPCR analysis. DNA was extracted from pellets obtained from a sedimentation assay using lettuce samples spiked with a range of *Toxocara* spp. egg concentrations and tested in duplicate. A sample was considered positive if there was detectable DNA at or before a cycle threshold of 38.

Sample name	Cq value	Result
5 egg spike	0.00	NEGATIVE
5 egg spike	0.00	NEGATIVE
10 egg spike	0.00	NEGATIVE
10 egg spike	0.00	NEGATIVE
20 egg spike	0.00	NEGATIVE
20 egg spike	36.58	POSITIVE
50 egg spike	35.10	POSITIVE
50 egg spike	34.33	POSITIVE
100 egg spike	35.06	POSITIVE
100 egg spike	35.91	POSITIVE
*T.canis* positive control	19.29	POSITIVE
*T.canis* positive control	19.51	POSITIVE
*T.cati* positive control	16.62	POSITIVE
*T.cati* positive control	16.42	POSITIVE
Negative control	0.00	NEGATIVE
Negative control	0.00	NEGATIVE

As the sedimentation assay was found to be the most successful method for recovering *Toxocara* spp. eggs from spiked lettuce samples, this method was used to test the vegetable samples obtained from the community gardens. Of the 82 vegetable samples analysed, *Toxocara* spp. eggs were identified by microscopy in two lettuce samples giving an overall prevalence of 2.4% (2/82) (95% CI = 1.3–3.5%), and specifically for lettuce (*N* = 31) a prevalence of 6.5% (2/31) (95% CI = 4.7–8.3%). Although not the primary focus of the study, one sample among the eight soil samples tested was also positive (one egg detected) and was not associated with a positive vegetable sample. The species of *Toxocara* egg observed could not be determined by light microscopy analysis due to morphological similarities present between species. qPCR testing of the pellets obtained did not yield any positive results. The number of vegetable and soil samples collected from each site and the numbers of positive samples are reported in Table 2.

**Table 2:** The quantity of each type of vegetable collected per community garden site is shown. The sources of the two positive lettuce samples are highlighted in bold. The availability of soil received from a site is also indicated. ‘Other’ samples included pak choi (*Brassica rapa subsp. Chinensis*), Swiss chard (*Beta vulgaris*) and garden mint (*Mentha spicata*).

Site	Lettuce (*Lactuca sativa*)	Spinach (*Spinacia oleracea*)	Spring onion (*Allium fistulosum*)	Celery (*Apium graveolens*)	Other	Total	Soil sample provided (n)
1	0	0	0	0	0	0	0
2	3	1	0	0	1	5	0
3	1	0	2	0	2	5	3
4	0	0	0	0	0	0	0
5	0	0	0	0	0	0	0
6	**4**	0	0	1	0	5	0
7	2	2	0	0	0	4	0
8	0	1	1	0	1	3	0
9	3	0	0	0	1	4	0
10	0	2	1	0	0	3	0
11	1	2	1	0	1	5	0
12	3	5	1	1	0	10	0
13	6	3	1	0	2	12	3
14	**5**	2	1	0	2	10	0
15	3	0	1	2	8	14	2
16	0	2	0	0	0	2	0
TOTAL	31	20	9	4	18	82	8

Anonymous questionnaire data were obtained from 77 plot holders donating their vegetables, which revealed that 88% (68/77) of respondents had seen a definitive host species or the excrement of a definitive host on their site; in both cases foxes and their faeces were most commonly reported. Furthermore, 29% (22/77) of respondents reported seeing all three host species at their community garden. A breakdown of definitive host species sightings and faecal observations is reported in Table 3. The association between seeing a definitive host and observing faeces on-site was found to be statistically significant (*P* < 0.0034).

**Table 3:** Data obtained from plot holder questionnaires (*N* = 77), with the numbers of respondents observing definitive host species and their faecal deposits in their community garden. DH = Definitive host.

	No. Plot holders	% Plot holders
Any DH species observed	62	81
Any DH faeces observed	52	68
DH or faeces observed	68	88
Fox seen on-site	52	68
Fox faeces	28	36
Cat seen on-site	38	49
Cat faeces	9	12
Dog seen on-site	38	49
Dog faeces	10	13
2 DH species observed	16	21
3 DH species observed	22	29
Unknown type of faeces seen	10	13

In addition, 62% of plot holders reportedly using some form of temporary cover over their crops, which were utilised in strategic ways over selected species of vegetables at certain times of year. In all cases, some part of their plot was available for definitive hosts to access at any time.

## 4 Discussion

Here we present the first report of *Toxocara* spp. egg contamination of vegetable produce grown in community gardens in Europe. Among the vegetables tested, both samples positive for *Toxocara* eggs were from lettuces (*Lactuca sativa*). The relatively low prevalence value of 2.4% obtained in this study for all vegetables combined is in line with other studies undertaken in different countries. For example, in Turkey the prevalence on salad vegetables was found to be 1.5%, with eggs detected on lettuce and parsley samples (Kozan et al., 2005). Similar values of 3.97% and 1.68% were obtained from ready-to-eat vegetables in Iran (Fallah et al., 2016; Rostami et al., 2016) and likewise, in Switzerland a prevalence of 2.55% was reported from lettuce samples (Guggisberg et al., 2020). In those studies, samples were predominantly obtained from markets rather than community gardens. The cultivation practices used to produce these vegetables was not specified, but even if they were grown within secure areas closed-off to the outside, one must also be open to the potential for contamination of vegetables with *Toxocara* spp. eggs via unfiltered or wastewater used for irrigation (Bowman, 2021).

Leafy vegetables such as lettuces have been reported to have higher susceptibility to contamination with *Toxocara* spp. eggs compared to non-leafy vegetables, possibly due to the folded nature of the leaves, which capture more soil during cultivation (Hajipour et al., 2021; Abougrain et al., 2010; Paller et al., 2021). This is consistent with the current study, where both positive vegetable samples were from lettuces. Due to a relatively cold spring season in the year of sampling, there was a delay in harvesting celery and spring onions, thus donations of these vegetable types were lower in comparison to lettuce and spinach. This limitation must be taken into consideration, and firm conclusions regarding lower risk of contamination of celery and spring onion cannot be drawn at this stage. In the USA, root vegetables were more likely to be contaminated with oocysts of the zoonotic protist of cat origin, *Toxoplasma gondii*, than leafy vegetables (Lilly and Webster, 2021), although leafy vegetables were commonly contaminated in Palestine (Dardona et al., 2021). The importance of vegetables as a source of infection in outbreaks of toxoplasmosis is increasingly recognised (Pinto-Ferreira et al., 2019), and deserves greater investigation also for toxocariasis.

Very few published studies have attempted to test the sensitivity of their method for detecting *Toxocara* eggs on vegetables (Guggisberg et al., 2020). Given the variety of approaches to testing vegetables for parasitic eggs, a comparison of published methods (Lalle et al. (2018), Malkamäki et al. (2019) and Hajipour et al., (2021)) was undertaken to establish the most successful technique for recovering *Toxocara* spp. eggs from vegetables. The sedimentation method was found to recover the highest number of eggs in this study. The poor recovery of *Toxocara* spp. eggs obtained using the homogenisation and sieving methods is highly relevant for future surveys of this nature. Moreover, although sedimentation yielded the highest number of eggs, this method recovered ≤2% of the eggs added by spiking. Thus, the current study and other studies utilising conventional egg-recovery approaches are likely to greatly underestimate the number of *Toxocara* spp. eggs available for human consumption.

Molecular testing to detect *Toxocara* spp. eggs on vegetables is not as commonly reported in the literature compared to conventional methods. The relative merits of microscopy and molecular detection have been evaluated for protist contaminants of leafy vegetables, along with implications for standardised food safety testing (Berrouch et al., 2020; Chalmers et al., 2022), but remain largely unexplored for *Toxocara* spp. In this study, to determine the threshold by which positive vegetable samples yield a positive qPCR result, lettuce samples were spiked with a series of *Toxocara* sp. egg concentrations followed by sedimentation recovery and molecular analysis. The lowest concentration resulting in a positive qPCR result was found to be 20 eggs, with samples considered to be positive if there was detectable DNA at or before a cycle threshold of 38, as per Tyungu et al. (2020). By comparison, in a recent study, Guggisberg et al. (2020) were able to detect 4 *Toxocara* eggs per 300 g of lettuce using molecular approaches, although in this case different primer sets were utilised compared to the current study, and conventional rather than qPCR was employed. Given the relatively low mass (100 g) of vegetable samples tested in this study, there may not have been sufficient quantities of eggs present within the sedimentation pellets obtained to meet the detection threshold. This could explain why none of the samples obtained from community gardens were positive on qPCR analysis Future studies of this nature may wish to use larger vegetable sample weights to maximise the chances of detecting *Toxocara* sp. by qPCR analysis.

The qPCR assay used in this study was able to differentiate between *T. canis* and *T. cati* DNA in the positive control samples, a finding which could be beneficial for future investigations to elucidate the animal source of eggs contaminating vegetable samples. The eggs used for spiking lettuce samples in this study were confirmed to be *T. canis* on qPCR testing, which was not surprising given the fox origin of the adult worms harvested, with *T. canis* being the most common species infecting this host type (Okulewicz, 2008).

The questionnaire data obtained show that sightings of definitive hosts are common in community gardens, in particular foxes. This finding is supported by a previous study undertaken in Bristol, UK (Saunders et al., 1997), which concluded that allotment sites were frequently inhabited by urban foxes. Moreover, Poulle et al. (2017) found that cat, fox and dog faeces were common in kitchen gardens in France, with some gardens disproportionately affected. In the current study, a relatively high number of respondents also reported observation of faecal deposits from foxes in their community garden, although recognition of the exact type of faeces observed might not be reliable. Collectively, these findings have important implications for food safety. Supply of *Toxocara* spp. eggs from domestic carnivores can be suppressed by regular anthelmintic treatment (Morgan et al., 2013). The feasibility of administering anthelmintics to wildlife species to reduce *Toxocara* infection and subsequent egg shedding, however, poses a much greater challenge. All participating sites had perimeter fencing and locked gates, but sightings demonstrated the ease with which animals, including foxes and cats, can traverse these boundaries. The intermittent use of covers such as plastic and netting over crops by plot holders may have some impact on the number of eggs in the soil and thus reduce vegetable contamination, but if there is any opportunity for animals to access soil for defecation, the persistence of infective *Toxocara* eggs in the environment for long periods needs to be considered, alongside the potential for contaminated water being used to irrigate these crops. In Kazakhstan, people who ate vegetables from gardens fertilized with dog faeces had an increased likelihood of testing positive for circulating anti-*Toxocara* antibodies (Torgerson et al., 2009). Vegetables from contaminated environments should be washed or cooked before being consumed, to avoid the risk of ingesting viable *Toxocara* eggs, while hand hygiene following contact with soil should also be observed. It should also be noted that absence of visible faeces does not rule out historical contamination and persistent infection risk from gardens, as confirmed for the zoonotic tapeworm of foxes and dogs, *Echinococcus multilocularis* (Da Silva et al., 2021), while cats tend to bury their faeces, concealing signs of elevated *Toxocara* hazard (Maciag et al., 2022).

Discovering *Toxocara* eggs within the soil used to cultivate vegetables in this study was not entirely unexpected given the presence of these eggs on vegetable crops. This finding is supported by similar studies undertaken in other countries, such as the Philippines (Paller et al., 2021) and Poland (Klapec and Borecka, 2012). Given the small number of soil samples tested in this pilot study, firm comparisons between the prevalence of eggs on vegetable produce and soil cannot be drawn. But finding one positive sample in a batch of eight soil samples shows that the eggs are present in the environment where vegetable crops are growing, and that they are being transferred to vegetable crops which are subsequently entering the food chain. The flow of *Toxocara* spp. from definitive hosts to food products warrants further investigation and could build upon the concept of this pilot study with a larger-scale evaluation of vegetable produce and upstream agricultural processes and contextual factors, including healthcare of domestic carnivores and biosecurity.

## 5 Conclusions

This proof-of-concept study revealed for the first time the presence of *Toxocara* spp. eggs on vegetables grown in community gardens and their surrounding soil. Definitive host species and their faecal deposits were commonly observed at participating sites. These findings highlight the importance of effective public health measures, including hand hygiene and vegetable washing, to reduce the risk of *Toxocara* transmission to humans via the cultivation and consumption of vegetables. The sedimentation method was found to be the most effective technique for the detection of *Toxocara* spp. eggs on lettuce samples and should be considered for future surveys of this nature. This small study paves the way to larger-scale investigations of *Toxocara* spp. egg contamination of field-grown vegetable produce, in order to inform food safety standards and safeguard consumer health.

## Funding

Sara Healy is funded by UK Research and Innovation, Biotechnology and Biological Sciences Research Council, through the FoodBioSystems Doctoral Training Programme.

## Declaration of Competing Interest

The authors declare that they have no known competing financial interests or personal relationships that could have appeared to influence the work reported in this paper.
